# Arterial stiffness and blood pressure improvement in aldosterone-producing adenoma harboring *KCNJ5* mutations after adrenalectomy

**DOI:** 10.18632/oncotarget.16269

**Published:** 2017-03-16

**Authors:** Chia-Hui Chang, Ya-Hui Hu, Yao-Chou Tsai, Che-Hsiung Wu, Shuo-Meng Wang, Lian-Yu Lin, Yen-Hung Lin, Fumitoshi Satoh, Kwan-Dun Wu, Vin-Cent Wu

**Affiliations:** ^1^ Division of Endocrinology and Metabolism, Department of Internal Medicine, Taipei Tzu Chi Hospital, The Buddhist Medical Foundation, Hualien, Taiwan; ^2^ Graduate Institute of Clinical Medicine, College of Medicine, National Taiwan University, Taipei, Taiwan; ^3^ Division of Urology, Department of Surgery, Taipei Tzu Chi Hospital, The Buddhist Medical Foundation, Hualien, Taiwan; ^4^ Division of Nephrology, Department of Internal Medicine, Taipei Tzu Chi Hospital, The Buddhist Medical Foundation, Hualien, Taiwan; ^5^ Division of Urology, Department of Surgery, National Taiwan University Hospital, Taipei, Taiwan; ^6^ Division of Cardiology, Department of Internal Medicine, National Taiwan University Hospital, Taipei, Taiwan; ^7^ Division of Clinical Hypertension, Endocrinology and Metabolism, Tohoku University, Graduate School of Medicine, Sendai, Japan; ^8^ Division of Nephrology, Department of Internal Medicine, National Taiwan University Hospital, Taipei, Taiwan; ^9^ TAIPAI, Taiwan Primary Aldosteronism Investigation (TAIPAI) Study Group, Taiwan

**Keywords:** KCNJ5 gene, arterial stiffness, PWV, TAIPAI, CAKS

## Abstract

The aim of this study was to show the effect of KCNJ5 mutational status on arterial stiffness in aldosterone-producing adenomas after adrenalectomy. Between February 2008 and January 2010, we prospectively enrolled 108 aldosterone-producing adenoma patients undergoing adrenalectomy. We conducted repeated measurements of pulse wave velocity at baseline, 6 months, and 12 months after adrenalectomy, grouped by KCNJ5 mutational status. Prognostic factors of arterial stiffness and risk for hypertension at 12 months after adrenalectomy were analyzed after propensity score matching in a 1:1 ratio. After matching for age, sex and body mass index, 88 patients were divided equally into KCNJ5-mutant and non-mutant groups. KCNJ5 mutational status was not an independent variable in either the generalized estimating equation model (*p* = 0.147) or the percentage change of brachial-ankle pulse wave velocity (*p* = 0.106). The generalized additive model smoothing plot showed that aldosterone-producing adenoma patients who carried the KCNJ5 mutation and were aged between 37 and 60 may have a hypertension recovery advantage. According to our observations during a 12-month follow-up after adrenalectomy, KCNJ5 mutational status was not associated with improvement in arterial stiffness.

## INTRODUCTION

Primary aldosteronism is one of the most common causes of endocrine hypertension [1, 2]. Dysregulation of excess aldosterone causes patients to be at high risk of refractory hypertension, severe hypokalemia or related cardiovascular morbidity and mortality [2–4].

The most frequent somatic mutation in aldosterone-producing adenoma (APA) occurs in the *KCNJ5* gene [5] and results in a loss of selectivity for potassium and the entry of sodium. The subsequent membrane depolarization and calcium mobilization activate CYP11B2 expression to stimulate overproduction of aldosterone [6–8]. Remarkably, according to a recent meta-analysis [9], the prevalence of *KCNJ5* somatic mutations is reported in 12–80% of APAs. The total prevalence of *KCNJ5* somatic mutations in APAs was 65.2% in a Japanese report [10], 76.8% in a Chinese report [11] and 59.5% in a Taiwanese report [12]. Evidence showed *KCNJ5*-mutant carriers were younger, had higher levels of preoperative aldosterone, more severe hypokalemia, and had a better hypertension prognosis after adrenalectomy than non-mutant carriers [9, 12–14]. Although it is well known that primary aldosteronism increases cardiovascular risk [15, 16], we are interested in knowing whether *KCNJ5*-mutant carriers have a higher risk of cardiovascular damage than non-mutant carriers.

Pulse wave velocity (PWV) has been documented as a useful method for evaluating the extent of arterial stiffness and coronary artery disease. It has been demonstrated that there is a significant inverse association between arterial stiffness and further cardiovascular disease [17–19]. A decrease in PWV is considered to correlate with improved hypertension or ideal cardiovascular health [20]. Although increases in left ventricular mass index (LVMI) and left ventricular hypertrophy (LVH) has been detected in *KCNJ5*-mutant carriers [21, 22], evidence evaluating the outcome of vascular status in those with *KCNJ5* somatic mutations after adrenalectomy was insufficient. In this cohort study, we conducted repeated measurements with multivariate adjustment for brachial-ankle pulse wave velocity (baPWV) at baseline, 6 months and 12 months after adrenalectomy to evaluate serial changes in arterial stiffness and hypertension in patients with and without *KCNJ5* somatic mutations.

## RESULTS

### Preoperative characteristics of the selected APA patients grouped by *KCNJ5* somatic mutations following propensity score matching

After 2 APAs with ATP1A1 mutation, 1 APA with ATP2B3 mutation, and 8 APAs with CTNNB1 mutation were excluded, a total of 108 patients (44 men and 64 women) clinically diagnosed with APA and undergoing adrenalectomy were enrolled in this study, including 61 *KCNJ5* somatic mutations and 47 wild type controls. The average age of the 108 patients was 49.3 ± 11.9 years and the average BMI was 25.0 ± 3.6 kg/m^2^. The median duration of hypertension was 5.0 (2.0–10.0) years. Table 1 summarizes the characteristics of the 108 APA patients grouped by *KCNJ5* somatic mutations before and after propensity score matching. Before matching, *KCNJ5*-mutant carriers were younger (*p* < 0.001) and had lower prevalence of dyslipidemia (*p* = 0.017), than non-mutant carriers. The *KCNJ5*-mutant group had significantly higher log-transformed plasma aldosterone concentration (PAC) (*p* = 0.039), aldosterone-renin ratio (ARR) (*p* = 0.041), higher estimated glomerular filtration rate (eGFR) (*p* < 0.001) and lower serum potassium level (*p* < 0.001) than non-mutant carriers. After matching by adjusting for age, sex and body mass index (BMI), 88 patients were equally divided into *KCNJ5*-mutant and non-mutant groups. The baseline characteristics were not significantly different between these groups, however the serum potassium levels were lower in the mutation carriers than their mutation-free counterparts (Table 1).

**Table 1:** Main characteristics of the APA patients grouped by KCNJ5 somatic mutations before and after propensity score matching

Variables	Before propensity score matching			After propensity score matching by age, sex, and BMI		
	KCNJ5-mutant group, *n* = 61	Non-mutant group, *n* = 47	*p*	KCNJ5-mutant group, *n* = 44	Non-mutant group, *n* = 44	*p*
**Age (years)**	44.9 ± 10.3	54.9 ± 11.6	< 0.001**	49.9 ± 7.0	53.6 ± 10.8	0.061
**Female (%)**	64	53	0.267	30	43	0.192
**BMI (kg/m^2^)**	25.0 ± 3.6	25.1 ± 3.8	0.875	24.4 ± 3.3	25.3 ± 3.8	0.266
**Heart rate(beat/min)**	71 ± 11	69 ± 14	0.450	70 ± 10	68 ± 14	0.565
**MBP (mmHg)**	120 ± 17	117 ± 15	0.275	119 ± 17	117 ± 16	0.682
**Diabetes (%)**	15	21	0.175	16	20	0.592
**Dyslipidemia (%)**	38	68	0.017*	39	62	0.082
**Sleep apnea syndrome (%)**	13	11	0.895	11	9	0.739
**HRT (%)**	5	4	0.882	7	5	0.674
**Smoking (%)**	10	13	0.456	5	11	0.269
**Pre-drug number**	1.9 ± 1.0	1.8 ± 0.7	0.896	1.8 ± 1.0	1.8 ± 0.8	1.000
**Post-drug number**	0.3 ± 0.6	0.5 ± 0.7	0.064	0.3 ± 0.7	0.5 ±0.6	0.255
**Duration of HTN(years)**	3.0 (2.0–10.0)	7.0 (2.0–15.0)	0.165	7.0 (2.0–13.0)	7.0 (1.25–10.0)	0.986
**PAC (ng/dL)**	60.4 (34.8–90.4)	38.8 (29.8–68.3)	0.045*	55.6 (32.8–82.9)	39.4 (29.9–71.2)	0.305
**Log (PAC)**	1.76 ± 0.28	1.65 ± 0.27	0.039*	1.72 ± 0.29	1.66 ± 0.27	0.284
**PRA (ng/ml/h)**	0.15 (0.02–0.45)	0.26 (0.06–0.44)	0.193	0.15 (0.05–0.42)	0.25 (0.07–0.43)	0.271
**ARR(ng/dl per ng/ml/h)**	426.9 (139.4–2325.8)	175.5 (78.0–738.8)	0.036*	333.4 (116.5–1435.7)	180.8 (80.7–724.2)	0.140
**Log (ARR)**	2.72 ± 0.75	2.42 ± 0.75	0.041*	2.64 ± 0.73	2.42 ± 0.74	0.161
**Serum potassium(mmol/l)**	3.2 ± 0.6	3.8 ± 0.6	< 0.001**	3.3 ± 0.6	3.8 ± 0.6	< 0.001**
**eGFR (CKD-EPI) (ml/min/1.73 m2)**	87.7 ± 23.5	69.5 ± 26.4	< 0.001**	81.8 ± 22.7	71.7 ± 25.6	0.054
**Baseline baPWV (cm/s)**	1514.3(1364.0–1723.9)	1599.8(1408.9–1794.0)	0.210	1553.8(1435.0–1822.6)	1562.4(1400.8–1778.8)	0.967

APA = aldosterone-producing adenoma; ARR = aldosterone-renin ratio; BMI = body mass index; baPWV = brachial-ankle pulse wave velocity; eGFR(CKD-EPI), estimated glomerular filtration rate using Chronic Kidney Disease Epidemiology Collaboration; HRT = Hormone replacement therapy; HTN = hypertension; Log (PAC) = log-transformed plasma aldosterone concentration; Log (ARR) = log-transformed aldosterone-renin ratio; MBP = mean blood pressure; Pre-drug number = preoperative antihypertensive drug number; Post-drug number = postoperative antihypertensive drug number; PAC = plasma aldosterone concentration; PRA = plasma renin activity.

Data are expressed as mean ± SD or median (25th–75th percentile).

*p < 0.05, **p < 0.01.

### Subsequent baPWV change after adrenalectomy

We used average baPWV to decrease possible measurement error. The coefficient of variation of average baPWV was 18.6% whereas 18.9% at right baPWV and 18.7% at left baPWV. The results of the generalized estimating equations (GEE) model indicated that throughout the study, neither the presence nor absence of *KCNJ5* mutations had any effect upon baPWV values (*p* = 0.147). After adjusting for baseline comorbidities, only age (*p* < 0.001) and baseline baPWV (*p* < 0.001) exhibited statistical significance for baPWV serial change. In respect to our result, baseline diabetes, dyslipidemia, sleep apnea syndrome, hormone replacement therapy, smoking and eGFR were not independent factors on improvement of arterial stiffness. After adrenalectomy, the results of diabetes, dyslipidemia, hormone replacement therapy, and smoking compared between mutant and non-mutant group were shown on Supplementary Tables 1 and 2.

**Table 2:** Risk factors for residual hypertension in APA patients at 12 postoperative months after propensity score matching

Variable	Cure, *n* = 60	Non-cure, *n* = 28	Odds ratio	95% CI	*p* value
Age (years)	50.3 ± 9.4	54.8 ± 8.0	1.057	1.002–1.114	0.042*
Female (%)	68	54			
BMI (kg/m^2^)	24.5 ± 3.5	25.7 ± 3.5			
Heart rate (beats/min)	68 ± 12	71 ± 13			
MBP (mmHg)	118 ± 16	119 ± 16			
Diabetes (%)	18	18			
Dyslipidemia (%)	50	57			
Sleep apnea syndrome (%)	8	14			
HRT (%)	7	4			
Smoking (%)	8	7			
Pre-drug number	1.9 ± 0.8	1.7 ± 1.0			
Duration of HTN (years)	4.0 (2.0–10.0)	10.0 (3.5–15.0)			
PAC (ng/dL)	41.7 (30.6–78.0)	45.2 (32.2–75.3)			
Log (PAC)	1.68 ± 0.28	1.71 ± 0.28			
ARR (ng/dl per ng/ml/h)	343.4 (99.2–1435.7)	171.2 (80.5–536.7)			
Log (ARR)	2.60 ± 0.77	2.37 ± 0.64			
Serum potassium (mmol/L)	3.5 ± 0.7	3.7 ± 0.6			
eGFR (CKD-EPI)(ml/min/1.73 m2)	79.7 ± 23.6	70.5 ± 25.9			
KCNJ5 somatic mutation (%)	57	36			
Baseline baPWV (cm/s)	1518.9 (1373.6–1777.4)	1635.0 (1473.4–1894.6)			

APA = aldosterone-producing adenoma; ARR = aldosterone-renin ratio; BMI = body mass index; baPWV = brachial-ankle pulse wave velocity; eGFR(CKD-EPI), estimated glomerular filtration rate using Chronic Kidney Disease Epidemiology Collaboration; HRT = Hormone replacement therapy; HTN = hypertension; Log (PAC) = log-transformed plasma aldosterone concentration; Log (ARR) = log-transformed aldosterone-renin ratio; MBP = mean blood pressure; Pre-drug number = preoperative antihypertensive drug number; PAC = plasma aldosterone concentration; PRA = plasma renin activity.

Data are expressed as mean ± SD or median (25th–75th percentile).

*p < 0.05.

**Figure 1:**
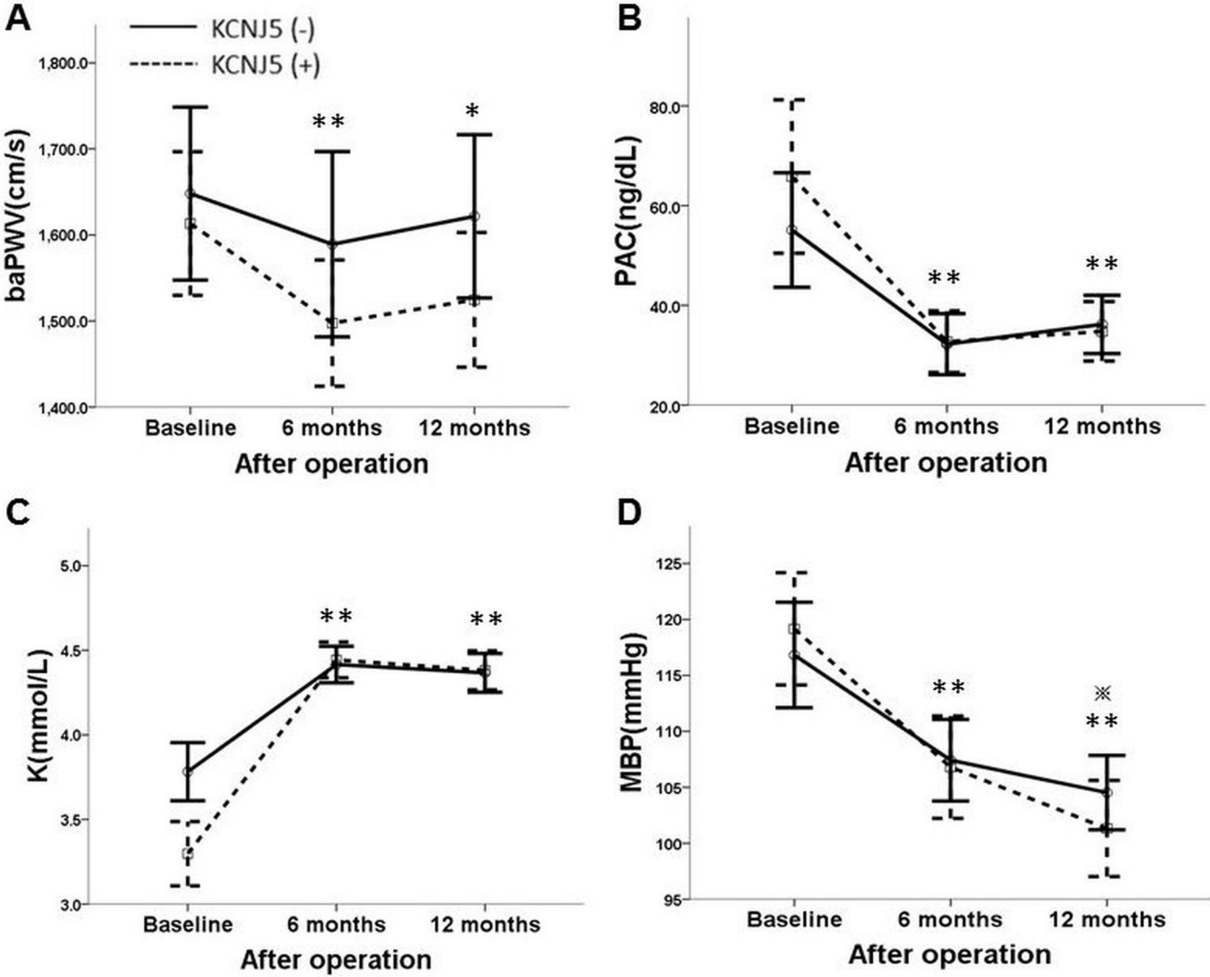
(**A**) The line chart shows repeated measurements of PWV grouped by *KCNJ5* somatic mutations. No interaction with *KCNJ5* mutational status and PWV during serial visits was detected. Only age and baseline PWV showed important roles for following PWV after adrenalectomy by GEE (*p* < 0.01). (**B**) Serial change of PAC grouped by *KCNJ5* somatic mutations. Mild rebound of PAC at 6 to 12 postoperative months was noted in both groups, but the restored serum aldosterone level was not significantly different at 6 months to 12 months after adrenalectomy. (**C**) Serial improvement of serum potassium level grouped by *KCNJ5* somatic mutations. Stable serum potassium level at 6 to 12 months after adrenalectomy is shown. (**D**) Serial improvement of MBP grouped by *KCNJ5* somatic mutations. The recovery of MBP at 6 to 12 months after adrenalectomy continued. PWV = pulse wave velocity; PAC = plasma aldosterone concentration; K = serum potassium level; MBP = mean blood pressure. *p < 0.05, **p < 0.01 vs. baseline among 88 patients; *p < 0.01 vs. 6 months among 88 patients.

**Figure 2:**
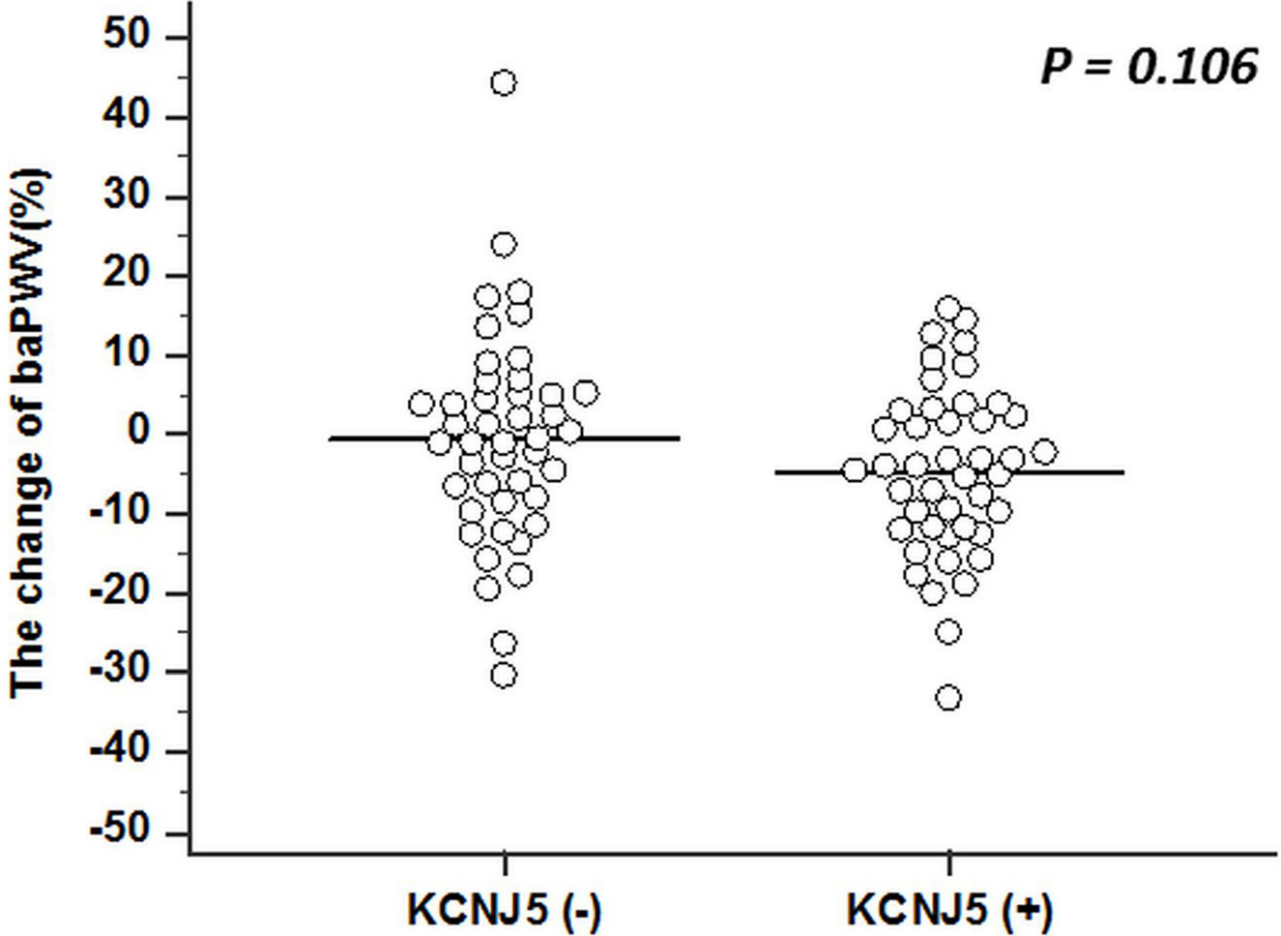
The dot plot shows the percentage change of PWV after 12 postoperative months, grouped by *KCNJ5* somatic mutations No statistically significant difference was detected between the two groups using independent *t* test. PWV = pulse wave velocity.

There was no further postoperative improvement of arterial stiffness at 6 to 12 months after adrenalectomy (Figure 1A) in both groups, with similar findings for aldosterone levels (Figure 1B) and serum potassium level (Figure 1C). In contrast, the mean blood pressure (MBP) improved gradually over 12 postoperative months (Figure 1D). The percentage change of baPWV at 12 months post operation, when plotted according to *KCNJ5* mutational status, showed that the mutation did not make a difference (*p* = 0.106) (Figure 2).

### The predictive factors for cure of hypertension and improvement of baPWV

**Figure 3:**
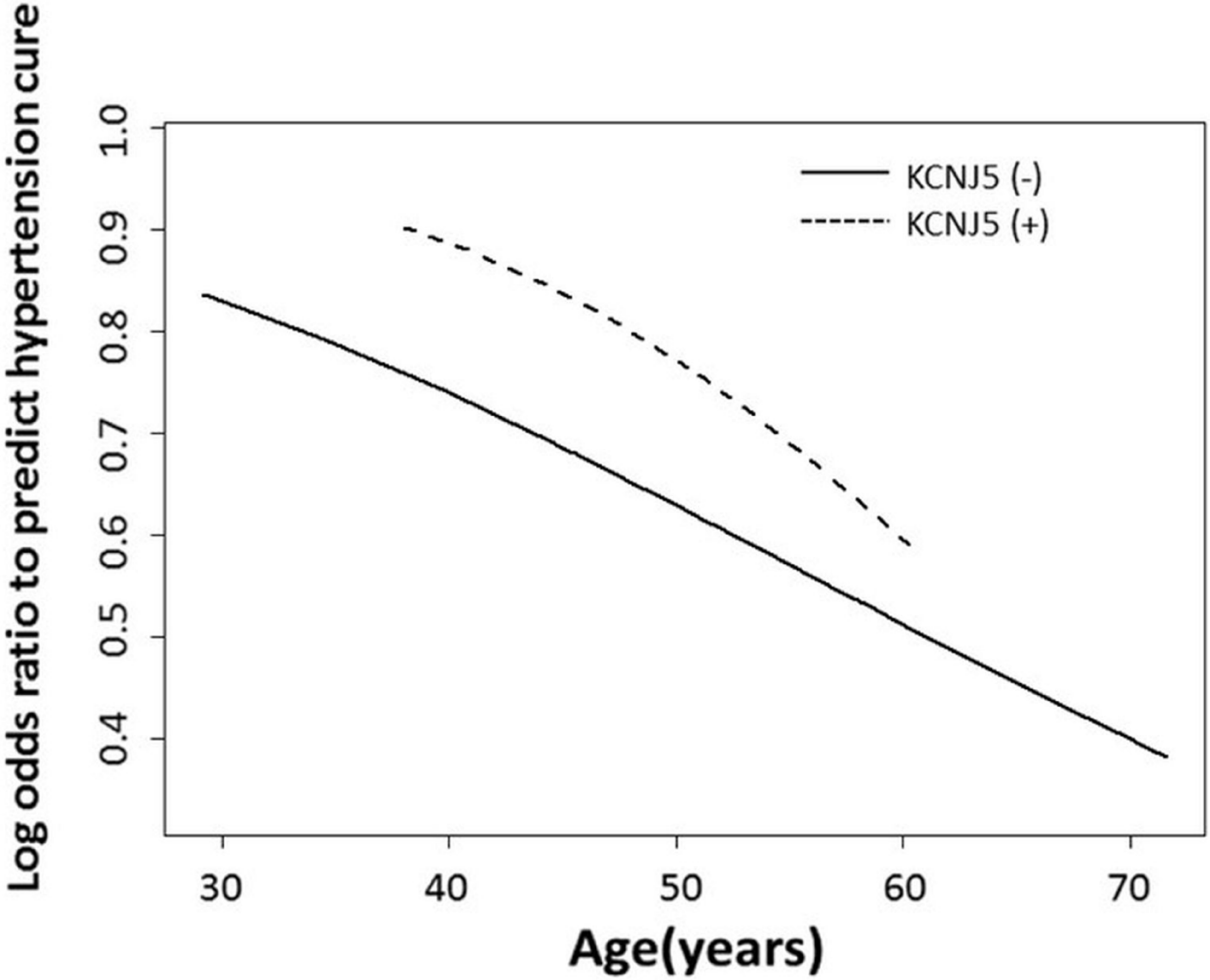
The GAM smoothing plot shows the log odds ratio to predict cure of hypertension with spline age, adjusted by BMI and stratified by *KCNJ5* somatic mutations The plot shows APA patients ranging from 37 to 60 years old, and indicates that *KCNJ5*-mutant carriers have an advantage of hypertension cure. APA = aldosterone-producing adenoma; BMI = body mass index; GAM = generalized additive model.

After a follow-up of 12 months, 60 of the 88 patients (68.2%) had been cured of hypertension. Only older age (OR = 1.057, 95% CI 1.002-1.114, *p* = 0.042) was predictive of postoperative residual hypertension (Hosmer–Lemeshow Goodness-of-Fit test, *p* = 0.886) (Table 2). However, the mutational status of *KCNJ5* was not a predictive factor for curing hypertension. The generalized additive model (GAM) smoothing plot (Figure 3) shows the log odds ratio to predict the cure of hypertension according to chronological age, stratified by *KCNJ5* mutational status. The plot showed that APA patients who were *KCNJ5*-mutant carriers and aged between 37 to 60 years old, were conferred an advantage in recovering from hypertension. The baPWV decreased from baseline 1553.8 cm/s (1414.8–1805.1 cm/s) to 1460.4 cm/s (1353.1–1684.0 cm/s) at 6 months after adrenalectomy (*p* = 0.001) and to 1532.5 cm/s (1364.5–1703.5 cm/s) at 12 months after adrenalectomy (*p* = 0.013). A low degree of arterial stiffness, identified as baPWV < 1400 cm/s [19], was observed at 12 postoperative months in 29 (33%) of the 88 patients. Both older age (OR = 1.074, 95% CI 1.002-1.151, *p* = 0.045) and higher baseline baPWV (OR = 1.006, 95% CI 1.002–1.009, *p* = 0.001) remained significantly associated with persistent arterial stiffness (baPWV > 1400 cm/s) at 12 postoperative months, as determined by logistic regression. (Hosmer-Lemeshow Goodness-of -Fit test, *p* = 0.459) (Table 3). In addition, the risk factors for high baPWV (> 1400 cm/s) before surgery was show on Supplementary Table 3. The GAM plot for the effect of age on log odds ratio to predict high baPWV (> 1400cm/s) after adrenalectomy with adjustment for baseline baPWV and comorbidities was shown on Supplementary Figure 2. The GAM plot for the effect of age on log odds ratio to predict hypertension cure with multivariate adjustments by using unmatched 108 APA patients were shown on Supplementary Figure 3.

**Table 3:** Risk factors for high PWV in APA patients at 12 postoperative months after propensity score matching

Variable	baPWV < 1400 (cm/s),*n* = 29	baPWV > 1400 (cm/s),*n* = 59	Odds ratio	95% CI	*p* value
**Age (years)**	46.4 ± 7.5	54.4 ± 8.9	1.074	1.002–1.151	0.045*
**Female (%)**	55	68			
**BMI (kg/m^2^)**	24.8 ± 4.4	24.9 ± 3.1			
**Heart rate (beats/min)**	71 ± 11	68 ± 13			
**MBP (mmHg)**	114 ± 11	120 ± 18			
**Diabetes (%)**	10	22			
**Dyslipidemia (%)**	47	51			
**Sleep apnea syndrome (%)**	7	12			
**HRT (%)**	3	8			
**Smoking (%)**	3	10			
**Pre-drug number**	1.9 ± 0.9	1.8 ± 0.9			
**Post-drug number**	0.2 ± 0.4	0.5 ± 0.7			
**Duration of HTN (years)**	3.0 (0.9–10.0)	10.0 (3.0–13.0)			
**PAC (ng/dL)**	62.4 (36.0–92.9)	38.8 (31.5–70.7)			
**Log (PAC)**	1.77 ± 0.27	1.65 ± 0.27			
**ARR (ng/dl per ng/ml/h)**	288.8 (97.3–2782.5)	205.2 (89.0–754.0)			
**Log (ARR)**	2.65 ± 0.79	2.47 ± 0.71			
**Serum potassium (mmol/L)**	3.4 ± 0.6	3.6 ± 0.6			
**eGFR (CKD-EPI)(ml/min/1.73 m2)**	77.3 ± 23.5	75.7 ± 27.1			
***KCNJ5* somatic mutation (%)**	59	46			
**Baseline baPWV (cm/s)**	1379.3 (1291.1–1511.3)	1636.3 (1504.9–1916.1)	1.006	1.002–1.009	0.001**

APA = aldosterone-producing adenoma; ARR = aldosterone-renin ratio; BMI = body mass index; baPWV = brachial-ankle pulse wave velocity; eGFR(CKD-EPI), estimated glomerular filtration rate using Chronic Kidney Disease Epidemiology Collaboration; HRT = Hormone replacement therapy; HTN = hypertension; Log (PAC) = log-transformed plasma aldosterone concentration; Log (ARR) = log-transformed aldosterone-renin ratio; MBP = mean blood pressure; Pre-drug number = preoperative antihypertensive drug number; Post-drug number = postoperative antihypertensive drug number; PAC = plasma aldosterone concentration; PRA = plasma renin activity.

Data are expressed as mean ± SD or median (25th–75th percentile).

*p < 0.05, **p < 0.01.

## DISCUSSION

In this study, adjusting for possible variables, we found *KCNJ5* mutational status was not associated with baseline baPWV or the improvement of baPWV at 12 months after adrenalectomy. Recently, a univariate paired comparison study reported *KCNJ5*-mutant APA patients had significant improvements in the baPWV at 6 to 12 months after surgery [21]. It was speculated that the result may be related to younger age APA patients who harbored *KCNJ5* mutations. Of note, Rossi *et al*. [22] and Kitamoto *et al*. [21] both reported that *KCNJ5*-mutant carriers had a greater left ventricle mass and LVH than non-mutant carriers, indicating more detrimental cardiac damage. Rossi et al. reported the feasibility of achieving regression of LVH is a greater extent in *KCNJ5* mutant carriers than non-mutant controls, likely because they started form a higher baseline LVMI. To discuss about the discrepancy among the previous studies and ours, we performed a time-varying study with multivariate adjustment, especially age, in order to explore the nature of *KCNJ5* mutational status rather than univariate comparison between mutant carriers and wild type controls. We have showed age is the point to predict the difference between *KCNJ5* mutant and non-mutant.

Based on our study with multivariate analysis, the GEE model revealed that age and baseline baPWV were responsible for improvement of arterial stiffness within 12 postoperative months, while an elevated serum aldosterone level at baseline was not an influential factor. Despite the higher preoperative levels of PAC in patients with the *KCNJ5* mutation, there were no differences in the extent of improvement in arterial stiffness when compared to non-mutants. Excessive aldosterone contributed to myocardium fibrosis, renal impairment, and vessel endothelium dysfunction [15, 23, 24]. Reduction in vascular damage appears to arise from the decrease in aldosterone concomitant with adrenalectomy, but not the patient’s *KCNJ5* mutational status. Indeed, the difference in the baPWV between studies was inconclusive, possibly due to the numerous factors that influence baPWV, such as race, age, blood pressure, heart rate, and duration of hypertension [25].

The benefit of surgery for the improvement of arterial stiffness was observed in both groups within the initial 6 months, and attenuating at 6 to 12 postoperative months (Figure 1A). We assumed the “rebound phenomenon” may imply the gradual recovery of suppressed counter-side adrenal gland or natural course of arterial stiffness. In line with this, both the restored serum aldosterone and potassium level do not significantly differ at 6 to 12 months after adrenalectomy (Figure 1B–1C), however, the MBP could further improve over 12 postoperative months. Based on our study, the ceiling effect in the improvement of arterial stiffness, restored serum aldosterone, and potassium level was shown at 6 months after adrenalectomy, while the improvement of MBP continued to 12 postoperative months (*p* = 0.004) (Figure 1A–1D). This implies that the damaged vascular wall could not be fully restored or need an even longer time to recover despite hypertension cure after adrenalectomy. Increased vascular smooth muscle cells length/volume is responsible for the increased medial volume and therefore to the vascular remodeling [26]. Hypertensive effect will augment the functional and/or structural abnormalities of the arterial wall. The maladapted vascular structure present before adrenalectomy has difficulty reaching full recovery within a short time, even with normalization of blood pressure [26]. Arterial wall dispensability remained deteriorated in well-controlled hypertensive subjects compared with normotensive subjects [27].

In a systematic review, the rate of hypertension cure after adrenalectomy varied from 20% to 72% [28]. After matching, the 68.2% recovery rate of our enrollees was in line with this success rate. Our result supported the fact that there are many risks accounting for the residual hypertension in APA patients, even after normalization of their aldosterone secretion and restored arterial stiffness. In our study, we found that only younger ages had benefits had benefits in curing hypertension. This study further adds strength to evaluate the predictors of baPWV <1400 cm/s, which represents a low risk of hypertension onset [19], and found that only older age and higher baseline baPWV showed important roles in persistent arterial stiffness. An increase in baPWV by 1 m/s corresponded with increases of 12% in total cardiovascular events, 13% in cardiovascular mortality, and 6% in all-cause mortality in the general population [29]. Effective treatments reported for lowering baPWV, which mitigates cardiovascular disease, include antihypertensive medications, lipid-lowering drugs, oral diabetic drugs, and weight loss [19]. Based on our study, we suggested patients with primary aldosteronism should control underlying hypertension more aggressively and undergo lifestyle modification to lower baseline baPWV.

Our results reinforce the view that patients with somatic mutations are more likely to cure of hypertension after adrenalectomy. According to our observations during a 12-month follow-up after adrenalectomy, neither PAC nor *KCNJ5* mutational status was associated with improvement in arterial stiffness. Previous reports showed mutation carriers and non-carriers had similar degrees of proteinuria and left ventricular hypertrophy after multivariate adjustment [22, 30]. Therefore, despite mutation carriers having an earlier onset of the disease, whether *KCNJ5* mutation translating into a unique cardiovascular phenotype still needs further study. However, we had demonstrated that those patients with *KCNJ5* somatic mutations have a lower level of pro-inflammatory status independent of chronological age, and are more likely to be cured postoperatively [12]. The mechanism to explain *KCNJ5* mutant carriers aged between 37 to 60 years old may have better hypertension prognosis is unclear. Whether the effect of aldosterone on fluid retention, endothelial dysfunction, vascular damage or arteriolosclerosis [31–33] also needs further investigation. Overall, it appears that the possibility of identifying APA patients who are at risk of developing vascular dysfunction early is attractive because the cardiovascular risks could be reversed and hypertension cured by further targeted management in the early stages of disease.

This is the first study to conduct a repeated measurement model at 6 to 12 postoperative months to evaluate the association between the *KCNJ5* mutational status and baPWV change in APA patients. A few limitations should be noted. Due to the study period being limited to 12 follow-up months, we could not further pursue the influence of the *KCNJ5* somatic mutations or the long-term improvement of baPWV over the 12 follow-up months. In addition, using the saline infusion test as the only confirmatory test likely excluded many cases of angiotensin-responsive APA [34, 35]. In the 108 cohorts, the tumor size on computed tomography (CT) ranged from 0.8 to 2.8 cm. The tumor size on histology ranged from 0.8 cm^3^ to 16.5 cm^3^. Using the mouse monoclonal antibody for CYP11B2 and H-E stain, we extracted the possibility of adenoma for study. However, it was possible that we misjudged the adenomas composed of different somatic mutations due to missing the tissue for DNA extraction.

In conclusion, our results provide evidence that during 12 months of follow-up of APA patients after adrenalectomy, *KCNJ5* mutational status was not associated with the improvement of arterial stiffness. Clinically, patients who are younger tend to have an advantage in being cured of hypertension after adrenalectomy.

## MATERIALS AND METHODS

### Ethics statement

All protocol and procedures complied with the standards of the Declaration of Helsinki. The study was approved by the institutional review board of the National Taiwan University Hospital (Taipei, Taiwan) (200611031R). All enrollees involved in this study provided signed and informed consent.

### Patient selection

All patients were registered in the Taiwan Primary Aldosteronism Investigation (TAIPAI) database between February 2008 and January 2010 [12, 36–39]. This prospective study enrolled patients diagnosed with APA and who had undergone adrenalectomy. For quality assurance, the database was constructed at two medical centers, their four affiliated hospitals, and two local hospitals in different cities in Taiwan. Before confirmatory tests were conducted, all antihypertensive medications were discontinued for at least 21 days. Diltiazem and/or doxazosin were administered to control markedly high blood pressure when required [40]. The study excluded patients who had clinically significant comorbid conditions, such as uncontrolled hypertension, New York Heart Classification (NYHC) III-IV congestive heart failure, stage 3 or higher chronic kidney disease ([GFR] < 60 mL/[min 1.73 m^2^], or diagnosed with cancer within the previous 5 years.

### Primary aldosteronism confirmation

All patients with a high ARR were confirmed to have primary aldosteronism by saline infusion test and subsequent imaging studies for subtype identification (Supplementary Figure 1). The diagnosis of APA was established in hypertensive patients with elevated ARR, TAIPAI score more than 60%, post-saline loading PAC > 10 ng/dl, evidence for lateralized disease by adrenal CT (*n* = 108), adrenal venous sampling (AVS) (*n* = 72) or NP-59 scintigraphy (*n* = 56), pathologically proven adenoma after adrenalectomy and subsequent biochemical or hypertension improvement (Supplementary Text 1).

Before AVS or NP-59 scintigraphy, antihypertensive drugs were discontinued or modification at least 21 days. In 72 unilateral APA patients, AVS was performed by our experienced radiologists without ACTH stimulation. Successful catheterization to each adrenal vein was confirmed based on the target to peripheral venous cortisol ratio greater than 2. Adrenal vein aldosterone to cortisol ratio (A/C ratio) was used to verify unilateral aldosterone hypersecretion. If A/C ratio between lesion and contralateral side greater than 2 with the phenomenon of contralateral suppression compared with peripheral vein, unilateral localization was confirmed. Some patients from our affiliated hospitals only had NP-59 adrenal scintigraphy. Adrenal scintigraphy with NP-59 SPECT/CT has documented as a useful tool for lateralization of excess aldosterone. Patients underwent noninvasive NP-59 SPECT/CT adrenocortical scintigraphy under 8-mg dexamethasone suppression daily for 1 week, and 1 mL of Lugol solution daily to protect thyroid. Scanning would be performed after NP-59 injection for 3 to 5 days. Unilateral predominant NP-59 uptake was confirmed to have aldosterone lateralization (Supplementary Table 1 and Supplementary Figure 1). In the other 36 patients with NP-59 SPECT/CT, all had unilateral increased NP-59 uptake compatible with CT image. After adrenalectomy, tumor part was identified by over expression of CYP11B2 mRNA or Immunohistochemistry. It was performed using mouse monoclonal antibody for CYP11B2 and rat monoclonal antibody for CYP11B1 [41, 42]. In all 108 patients, precise detection of localization was confirmed following pathologic reports and postsurgical response including hypertension or biochemical improvement.

### Definition of hypertension outcome, clinical and biochemical parameters

Postoperatively, patients were asked to self-monitor their blood pressure once a month for the first three months, and subsequently every three months for up to one year. Patients were defined as cured with a resolution of hypertension if 75% of their home recorded blood pressure measurements decreased to < 140/90 mmHg without any antihypertensive medications at least one year after adrenalectomy. Those that did not meet these criteria fell into the ‘non-cure’ group [12]. We collected patients’ records for the following clinical and biochemical parameters: age, gender, BMI, heart rate, blood pressure, serum potassium levels, PAC, plasma renin activity (PRA) and histopathology findings. Preoperative blood pressure was measured at the first visit using a sphygmomanometer for evaluation of secondary hypertension in the outpatient department [12, 36].

### Sequencing of the *KCNJ5* gene

All specimens of adrenal tumors and a sample of normal adrenal cortex were frozen and stored at −72°C in liquid nitrogen. DNA was extracted using a QIAamp DNA mini kit (Qiagen, Hilden, Germany). PCR solutions were prepared according to the manual for Platinum Taq high fidelity (Invitrogen, Carlsbad, CA, USA) with a final volume of 50 μl. After DNase I treatment, 500 ng of total RNA were reverse-transcribed using Moloney murine leukemia virus reverse transcriptase (M-MLV RT) (Promega, Madison, WI, USA) and random hexamers (Promega, Madison, WI, USA) according to the manufacturer’s instructions. The entire coding sequence (exons 2–3) and flanking regions of *KCNJ5* were amplified and sequenced using gene-specific primers. Direct sequencing of PCR products was performed using The BigDye Terminator v3.1 cycle sequencing kit (Applied Biosystems, Foster City, USA) with a 3730 DNA Analyzer (Applied Biosystems, Foster City, CA, USA). Standard protocol of sequencing in TAIPAI followed that which had been previously reported [12].

### Measurement of bilateral baPWV

BaPWV, the distance between brachial and ankle, was measured with an automatic waveform analyzer (Colin VP-2000, Omeron Inc., Japan). The participants were asked to rest, keeping calm and quiet, for at least 15 minutes in a supine position before taking the measurements in the morning. This machine simultaneously recorded the waveforms of bilateral brachial and carotid arteries, phonocardiograms, and electrocardiogram [37, 43]. To assess arterial stiffness in all participants, measurements of bilateral baPWV were repeated at baseline, 6 months and 12 months after operation. At each examination, patients received two repeated measurements, separated by a 5 minute interval. A total of four values of baPWV were recorded in every patient. Average baPWV values were used for data analyses.

### Statistical analysis

First, we calculated the power of the single locus analysis using a genetic power calculator (http://pngu.mgh.harvard.edu/~purcell/gpc/) [44]. Given the 12% prevalence of primary aldosteronism in hypertension patients [1, 45], the calculated power was 0.847 with percentages of having at least one variant allele from 0.047 to 0.318 to detect an OR of 1.8 with a two-sided α error of 0.05. Estimation of sample size was based on previous reports of patients with or without *KCNJ5* mutation and those who were associated with significantly different baPWV [21]. Assuming a type I error (α) as 0.01 and Type 2 error (β) as 0.10, at least 42 patients in each group were necessary to show a calculated 90% power.

*KCNJ5* somatic mutations and wild type APA patients without *ATP1A1*, *ATP2B3*, *CTNNB1* and *CACNA1D* mutations were matched by propensity score in a 1:1 ratio, adjusting for age, sex and BMI. Kolmogorov-Smirnov test or Shapiro-Wilk test were used to test for normal distribution. Results were expressed as mean and standard deviation if normally distributed, or median and interquartile range if not. Log transformation would be applied for skewed distribution, such as PAC and ARR. The difference of preoperative variables between the *KCNJ5*-mutant and non-mutant groups were calculated by using independent *t* test or Mann-Whitney *U* test depending on whether the distribution was normal or not. Chi-square tests was used for the comparison of two proportions. The paired *t*-test or Wilcoxon signed-rank test was used to compare preoperative and postoperative variables depending on their normal distribution or non-normality, too.

To explore differences among measurements of baPWV at baseline, 6 months and 12 months after operation, grouped by *KCNJ5* somatic mutations, we fitted the marginal linear regression models to the repeatedly measured responses using the GEE method [46]. Adjustments were made for age, sex, BMI, heart rate, MBP, duration of hypertension, log transformation of PAC, and visits in this GEE model. The significance level for entry (SLE) and for stay (SLS) were set conservatively at 0.15. The advantage of GEE is that the model is efficient in achieving higher power with small sample sizes and it makes full use of lower numbers of repeated measurements in both complete and missing data [47].

The percentage change of baPWV grouped by *KCNJ5* somatic mutations after operation was estimated using independent *t* test because of their normal distribution. Logistic regression with a stepwise method was also used to predict the prognostic factors of complete hypertension cure and baPWV < 1400 cm/s. The Hosmer–Lemeshow Goodness-of-Fit test was applied to assess the fitted multiple logistic regression model. To evaluate the effect of age on the success for cure of hypertension, we adopted a GAM grouped by the *KCNJ5* mutational status, with adjustment for baseline comorbidities. This method also grants adjustments for possible nonlinear effects of continuous variables [34, 48]. A vector generalized additive model (VGAM) package was used, with the VGAM function set to default values for smoothing parameters, to fit GAM for the binary responses in R software. The result was shown as a function curve with values of the log odds ratio.

Statistical significance was defined as *p* < 0.05. Statistical analyses were performed with SPSS software, version 22.0 (IBM Corp. Released 2013. IBM SPSS Statistics for Windows, Version 22.0. Armonk, NY: IBM Corp), MedCalc Statistical Software version 16.8.4 (MedCalc Software bvba, Ostend, Belgium; https://www.medcalc.org; 2016) and R software, version 2.8.1 (Free Software Foundation, Inc., Boston, MA, U.S.A.).

## SUPPLEMENTARY MATERIALS FIGURES AND TABLES


