# Supplementary material for: Arterial stiffness and blood pressure improvement in aldosterone-producing adenoma harboring KCNJ5 mutations after adrenalectomy
Source: Oncotarget. 2017 Mar 16;8(18):29984–95. doi: 10.18632/oncotarget.16269 (PMC5444719; doi:10.18632/oncotarget.16269)
Supplement: Supplementary file 1 [file oncotarget-08-29984-s001.pdf]

Arterial stiffness and blood pressure improvement in aldosterone-producing adenoma harboring *KCNJ5* mutations after adrenalectomy

Supplementary Materials

MATERIALS AND METHODS

Enrolled hospitals

This study included two medical centers (National Taiwan University Hospital (NTUH), Taipei; Taipei University Hospital, Taipei), four metropolitan hospitals (Cardinal Tien Hospital, New Taipei City; Taipei Tzu Chi Hospital, New Taipei City; Yun- Lin Branch of NTUH, Douliou City; Tao-Yuan Hospital, Taoyuan City), and two local hospitals (Hsin-Chu Branch of NTUH, Hsin-Chu City; Zhongxing Branch of Taipei City Hospital, Taipei) [1, 2].

The standard protocol to identify aldosteronism

The diagnosis of aldosteronism was established in hypertensive patients on the basis of the following criteria [3–6]:(Supplementary Figure 1)

Confirmation

Fulfillment of the following three conditions confirms a diagnosis of aldosteronism: (1) autonomous excess aldosterone production evidenced with an ARR > 35; (2) a TAIPAI score larger than 60% [7]; (3) post-saline loading PAC > 10 ng/dl, or PAC/PRA> 35 (ng/dL)/(ng/mL/h) shown in a post capotopril/ losartan test, or PAC>6 ng/dL indicated by a fludrocortisone suppression test [6]. (Abbreviations: PAC, plasma aldosterone concentration; PRA, plasma renin activity)

Lateralization

APA is identified on the basis on the following four conditions: (1) autonomous excess aldosterone production evidenced with an ARR > 35, a TAIPAI score larger than 60% [7], and post-saline loading PAC > 10 ng/dl; (2) adenoma evidenced with a CT scan for pre-operative evaluation [6]; (3) lateralization of aldosterone secretion at AVS or during dexamethasone suppression NP-59 SPECT/CT [8]; (4) pathologically proven adenoma after an adrenalectomy for those with operations, and subsequent emergence of either a cure pattern of hypertension without anti-hypertensive agents or improvement in hypertension, potassium, PAC, and PRA [5, 6, 9].

Idiopathic hyperaldosteronism (IHA) is distinguished on the basis on the following four criteria: (1) autonomous excess aldosterone production evidenced with an ARR > 35, a TAIPAI score larger than 60% [7], and post-saline loading PAC > 10 ng/dl (2) evidence of bilateral diffuse enlargement indicated by a CT scan for pre-operative evaluation; (3) non-lateralization of aldosterone secretion at AVS or during dexamethasone suppression NP-59 SPECT/CT [8]; (4) evidence of diffuse cell hyperplasia reported in following pathology studies for those with operations.

REFERENCES

1. Wu VC, Hu YH, Wu CH, Kao CC, Wang CY, Yang WS, Lee HH, Chang YS, Lin YH, Wang SM, Chen L, Wu KD; TAIPAI Study Group. Administrative data on diagnosis and mineralocorticoid receptor antagonist prescription identified patients with primary aldosteronism in Taiwan. J Clin Epidemiol. 2014; 67:1139–1149.
2. Wu VC, Lo SC, Chen YL, Huang PH, Tsai CT, Liang CJ, Kuo CC, Kuo YS, Lee BC, Wu EL, Lin YH, Sun YY, Lin SL, et al. Endothelial progenitor cells in primary aldosteronism: a biomarker of severity for aldosterone vasculopathy and prognosis. J Clin Endocrinol Metab. 2011; 96:3175–3183.
3. Sechi LA, Novello M, Lapenna R, Baroselli S, Nadalini E, Colussi GL, Catena C. Long-term renal outcomes in patients with primary aldosteronism. JAMA. 2006; 295: 2638–2645.
4. Wu VC, Chang HW, Liu KL, Lin YH, Chueh SC, Lin WC, Ho YL, Huang JW, Chiang CK, Yang SY, Chen YM, Wang SM, Huang KH, et al. Primary aldosteronism: diagnostic accuracy of the losartan and captopril tests. Am J Hypertens. 2009; 22:821–827.
5. Kuo CC, Wu VC, Huang KH, Wang SM, Chang CC, Lu CC, Yang WS, Tsai CW, Lai CF, Lee TY, Lin WC, Wu MS, Lin YH, et al. Verification and evaluation of aldosteronism demographics in the Taiwan primary aldosteronism investigation group (TAIPAI Group). J Renin Angiotensin Aldosterone Syst. 2011; 12:348–357.
6. Chao CT, Wu VC, Kuo CC, Lin YH, Chang CC, Chueh SJ, Wu KD, Pimenta E, Stowasser M. Diagnosis and

management of primary aldosteronism: an updated review. Ann Med. 2013; 45:375–383.

7. Wu VC, Yang SY, Lin JW, Cheng BW, Kuo CC, Tsai CT, Chu TS, Huang KH, Wang SM, Lin YH, Chiang CK, Chang HW, Lin CY, et al. Kidney impairment in primary aldosteronism. Clin Chim Acta. 2011; 412:1319–1325.

8. Yen RF, Wu VC, Liu KL, Cheng MF, Wu YW, Chueh SC, Lin WC, Wu KD, Tzen KY, Lu CC; TAIPAI Study Group. 131I-6beta-iodomethyl-19-norcholesterol SPECT/CT for primary aldosteronism patients with inconclusive adrenal venous sampling and CT results. J Nucl Med. 2009; 50:1631–1637.

9. Wu VC, Chao CT, Kuo CC, Lin YH, Chueh SJ, Wu KD. Diagnosis and management of primary aldosteronism. Acta Nephrologica. 2012; 26:111–120.

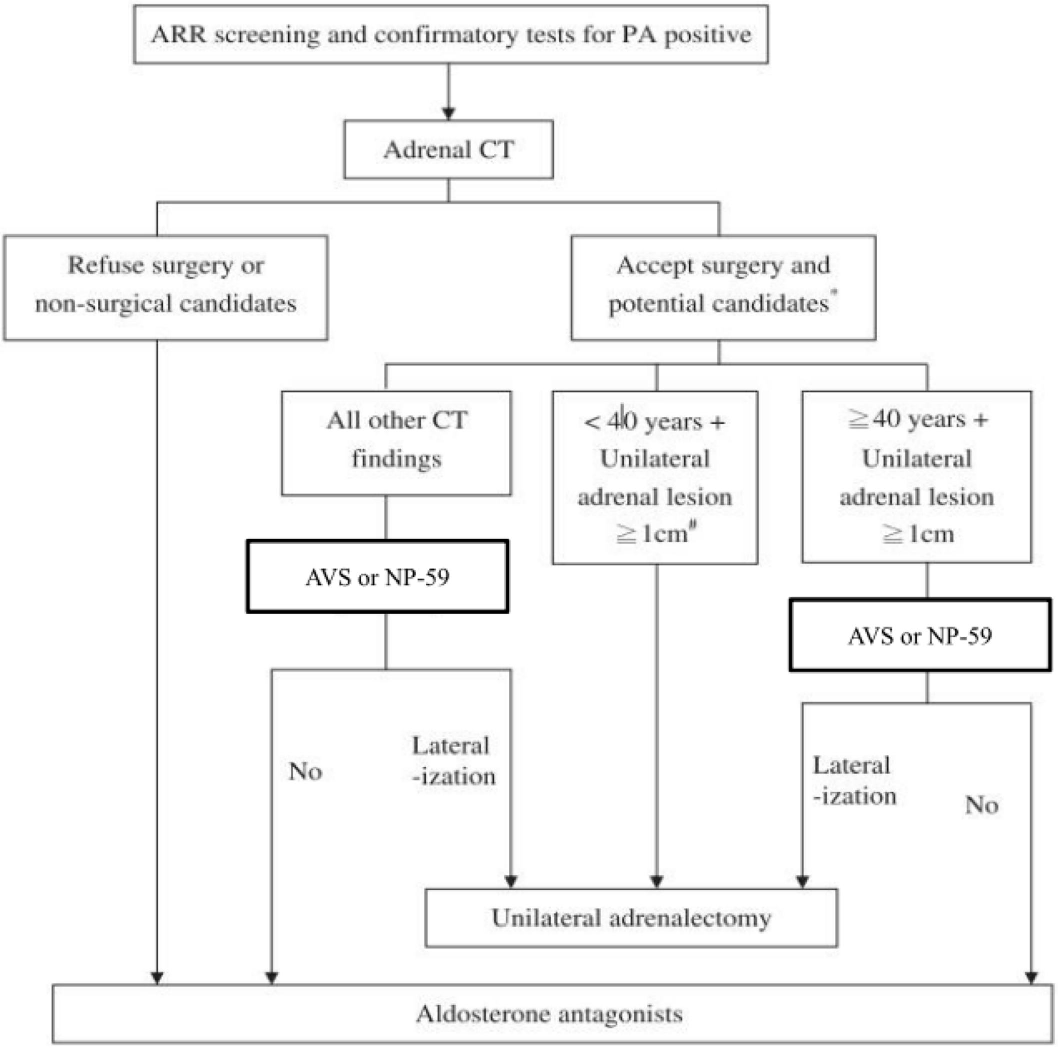

Supplementary Figure 1: The subtype-differentiating protocol of the TAIPAI group [5, 6, 9]. Abbreviations: AVS, adrenal venous sampling; APA, aldosterone-producing adenomas; ARR, aldosterone-to-renin ratio; CT, computed tomography; NP-59- SPECT, I-131-6-beta-iodomethyl-19-norcholesterol single-photon emission computed tomography.

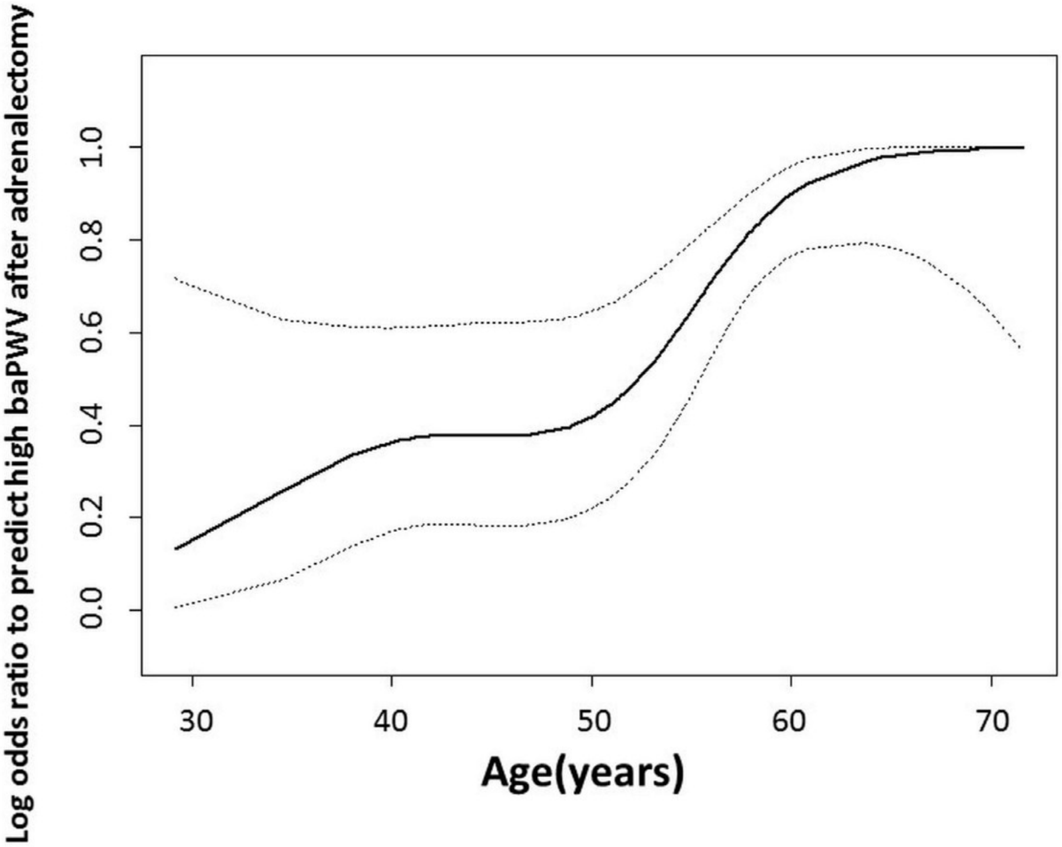

Supplementary Figure 2: The GAM plot for the effect of age on log odds ratio to predict. High baPWV (>1400 cm/s) after adrenalectomy with adjustment for baseline baPWV and comorbidities. The baPWV augmented against the chronological age. Abbreviations: GAM = generalized additive model; baPWV = brachial-ankle pulse wave velocity.

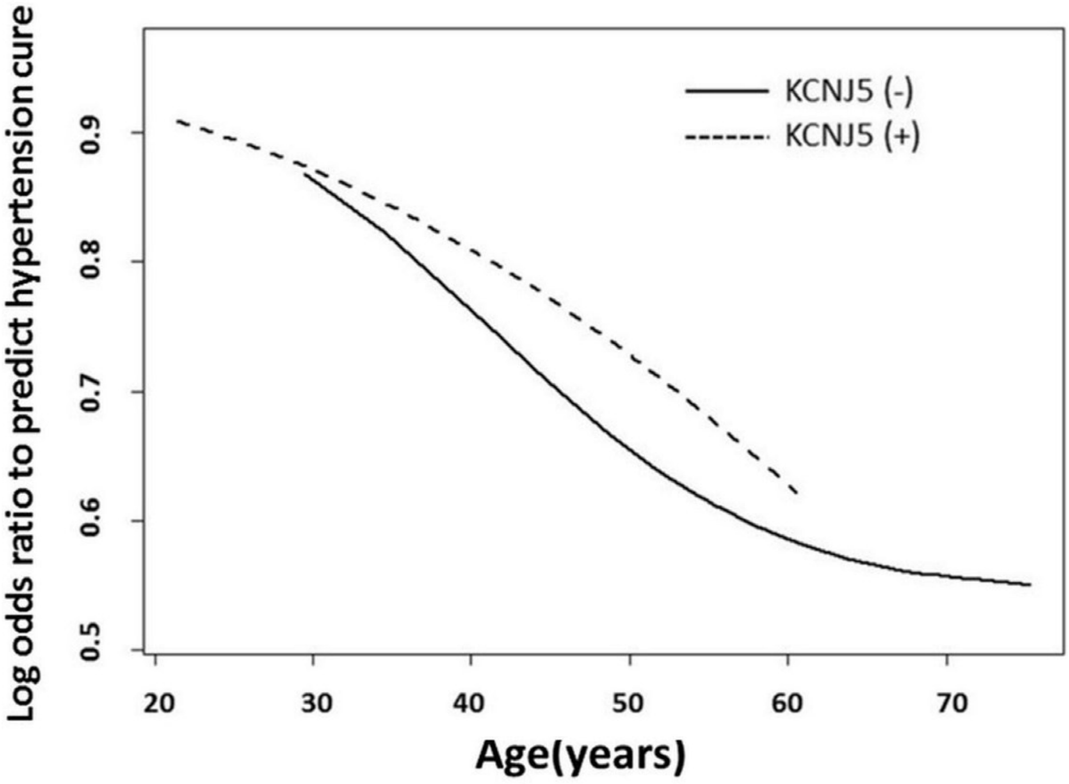

Supplementary Figure 3: The GAM plot for the effect of age on log odds ratio to predict. Hypertension cure with multivariate adjustments by using unmatched 108 APA patients showed similar result as Figure 3. *KCNJ5*-mutant group may have an advantage of hypertension cure than non-mutant group between 30 to 60 years old. Abbreviations: GAM = generalized additive model.

Supplementary Table 1: Univariate comparison between mutant and non-mutant group at 12 postoperative months

	After adrenalectomy		
**Variables**	***KCNJ5*-mutant group, *n* = 44**	**Non-mutant group, *n* = 44**	***p***
**Diabetes (%)**	11	18	0.386
**Dyslipidemia (%)**	34	55	0.058
**HRT (%)**	5	2	0.674
**Smoking (%)**	5	11	0.162

Abbreviations: HRT = hormone replacement therapy.

Supplementary Table 2: Paired sample test before and after adrenalectomy

	***KCNJ5*-mutant group, *n* = 44**			**Non-mutant group, *n* = 44**		
**Variables**	**Before adrenalectomy**	**After adrenalectomy**	***p***	**Before adrenalectomy**	**After adrenalectomy**	***p***
**Diabetes (%)**	16	11	ns	20	18	ns
**Dyslipidemia (%)**	39	34	ns	62	55	ns
**HRT (%)**	7	5	ns	5	2	ns
**Smoking (%)**	5	5	ns	11	11	ns

Abbreviations: HRT = hormone replacement therapy; ns = non-significance.

Supplementary Table 3: Risk factors for high baPWV in APA patients at baseline before surgery (Logistic regression with stepwise method)

Variables	baPWV < 1400 (cm/s), *n* = 20	baPWV > 1400 (cm/s), *n* = 68	O.R.	95% CI	*p*
Age(years)	45.3±6.9	53.6±9.0	1.15	1.058–1.243	< 0.001
MBP(mmHg)	110±10	120±17	1.07	1.018–1.129	0.009
Pre-drug number	1.6±0.8	1.9±0.9	2.35	1.062–5.199	0.035

Abbreviations: APA = aldosterone-producing adenoma; baPWV = brachial-ankle pulse wave velocity; MBP = mean blood pressure; Pre-drug number = preoperative drug number.
